# Burned-Out Autoimmune Disease: A Multicenter Case Series Revealing a Distinct Clinical Phase and Its Implications for Immunosuppressive Therapy

**DOI:** 10.7759/cureus.110070

**Published:** 2026-06-01

**Authors:** Ibrahim Saleh, Amjad Alkadi, Saleh Salman, Mostafa Abdelaty, Emad Wissa

**Affiliations:** 1 Rheumatology, Ministry of Health of Kuwait, Kuwait City, KWT; 2 Medicine/Cardiology, Aleppo University Hospital, Aleppo, SYR; 3 Internal Medicine, Al-Sabah Hospital, Kuwait City, KWT; 4 Respiratory Medicine, Al-Sabah Hospital, Kuwait City, KWT

**Keywords:** autoimmune disease, axial spondyloarthritis, burned-out disease, esrd, immunosuppression, lupus nephritis, vasculitis

## Abstract

Background and objective

Distinguishing active inflammation from irreversible organ damage in autoimmune diseases remains a major clinical challenge and may result in the inappropriate continuation of immunosuppressive therapy. The concept of “burned-out” autoimmune disease, in which inflammatory activity subsides following irreversible tissue damage, remains underrecognized. This study aimed to describe the burned-out phase across multiple autoimmune diseases and to propose a cross-disease clinical framework to differentiate active inflammation from irreversible damage.

Methods

We conducted a multicenter case series involving seven patients who were managed across tertiary care hospitals in Kuwait. The diseases included were lupus nephritis (n = 3), antineutrophil cytoplasmic antibody (ANCA)-associated vasculitis (n = 1), polyarteritis nodosa-like vasculitis (n = 1), and axial spondyloarthritis (n = 2). Clinical, laboratory, imaging, and histopathological data were collected and analyzed.

Results

All patients demonstrated clear evidence of prior active autoimmune disease followed by progression to irreversible organ damage, including end-stage renal disease (ESRD), vascular ischemia, and structural ankylosis. Subsequently, sustained clinical and serological quiescence was observed despite minimal or no immunosuppressive therapy. Continued immunosuppression in one case was associated with a fatal opportunistic infection. Disease activity during follow-up was retrospectively assessed using disease-specific validated measures - including the Birmingham Vasculitis Activity Score (BVAS)-based assessment for vasculitis, the Systemic Lupus Erythematosus Disease Activity Index (SLEDAI)-based serological assessment for lupus nephritis, and Axial Spondyloarthritis Disease Activity Score (ASDAS)/Assessment of Spondyloarthritis International Society (ASAS)-based assessment for axial spondyloarthritis - all of which supported inactive disease states in burned-out cases.

Conclusions

Burned-out autoimmune disease represents a clinically relevant but underrecognized phase across multiple conditions. Failure to distinguish this state from active inflammation may result in unnecessary and potentially harmful immunosuppressive therapy. Our findings support the need for a structured clinical approach to differentiate irreversible damage from ongoing disease activity. Further prospective studies are required to validate this concept and refine its clinical application.

## Introduction

Autoimmune diseases are characterized by immune-mediated inflammation targeting specific tissues, often following a relapsing-remitting course. Clinical decision-making is typically guided by the identification of ongoing inflammatory activity using clinical features, laboratory markers, imaging, and histopathology. However, in advanced stages of these diseases, irreversible structural damage may predominate while inflammatory activity diminishes or disappears [1,2]. This transition, referred to as “burned-out” autoimmune disease, represents a clinically important yet underrecognized state. Persistent symptoms such as pain, organ dysfunction, or functional limitation may reflect structural damage rather than active inflammation. Misinterpretation of these findings can lead to the unnecessary continuation or escalation of immunosuppressive therapy, exposing patients to significant risks, including infection, malignancy, and drug toxicity [1-3].

This phenomenon has been described in individual disease contexts. In antineutrophil cytoplasmic antibody (ANCA)-associated vasculitis, progression to end-stage renal disease (ESRD) is associated with reduced relapse rates, while infection-related mortality becomes a primary concern [4,5]. In axial spondyloarthritis, structural ankylosis may persist in the absence of active inflammation, limiting the benefit of biologic therapy and highlighting the need for objective inflammatory evidence before treatment escalation [6,7]. Similarly, in lupus nephritis, chronic glomerulosclerosis and ESRD may be associated with reduced immunological activity following extensive tissue destruction [8,9].

Despite these observations, a unified cross-disease framework to define and identify burned-out autoimmune disease is lacking. This gap has important implications for clinical decision-making, particularly in determining when immunosuppressive therapy should be de-escalated or discontinued. Although similar observations have been described in individual autoimmune diseases, this phenomenon remains underrecognized across rheumatic conditions. In this study, we present a multicenter case series illustrating the burned-out phenomenon and propose a practical cross-disease framework for distinguishing active inflammation from irreversible damage.

## Materials and methods

Study design and methodology

This retrospective multicenter case series was conducted across tertiary care hospitals in Kuwait between January 2024 and March 2026. Consecutive cases fulfilling predefined eligibility criteria were included.

Inclusion Criteria

The inclusion criteria were (1) previously confirmed autoimmune disease based on accepted clinical, serological, histopathological, or imaging criteria; (2) documented prior active inflammatory disease; (3) progression to irreversible organ damage such as ESRD, vascular infarction, fibrosis, or ankylosis; and (4) absence of objective evidence of ongoing inflammatory activity during follow-up.

Exclusion Criteria

The exclusion criteria were (1) insufficient clinical documentation; (2) absence of a confirmed autoimmune diagnosis; (3) active infection mimicking inflammatory disease activity; and (4) inadequate follow-up data to assess disease quiescence.

Cases were identified using consecutive sampling from rheumatology and internal medicine services. The study included cases collected from three tertiary care centers in Kuwait. Disease activity measures (including the Birmingham Vasculitis Activity Score (BVAS), the Systemic Lupus Erythematosus Disease Activity Index (SLEDAI), and Axial Spondyloarthritis Disease Activity Score (ASDAS)/Assessment of Spondyloarthritis International Society (ASAS)-based assessments) were retrospectively interpreted using documented clinical, laboratory, and imaging findings available in the medical records by the treating rheumatology teams. Potential confounding factors, including chronic infection, diabetic vascular disease, atherosclerosis, medication-related complications, and irreversible structural damage, were clinically assessed during follow-up evaluation.

Descriptive analysis was performed. Due to the small sample size and exploratory nature of the study, no inferential statistical comparisons were conducted. This study was conducted in accordance with the Declaration of Helsinki. According to local institutional policy, formal IRB/IEC approval was waived because of the retrospective anonymized non-interventional design. No identifiable patient information was included.

**Figure 1:**
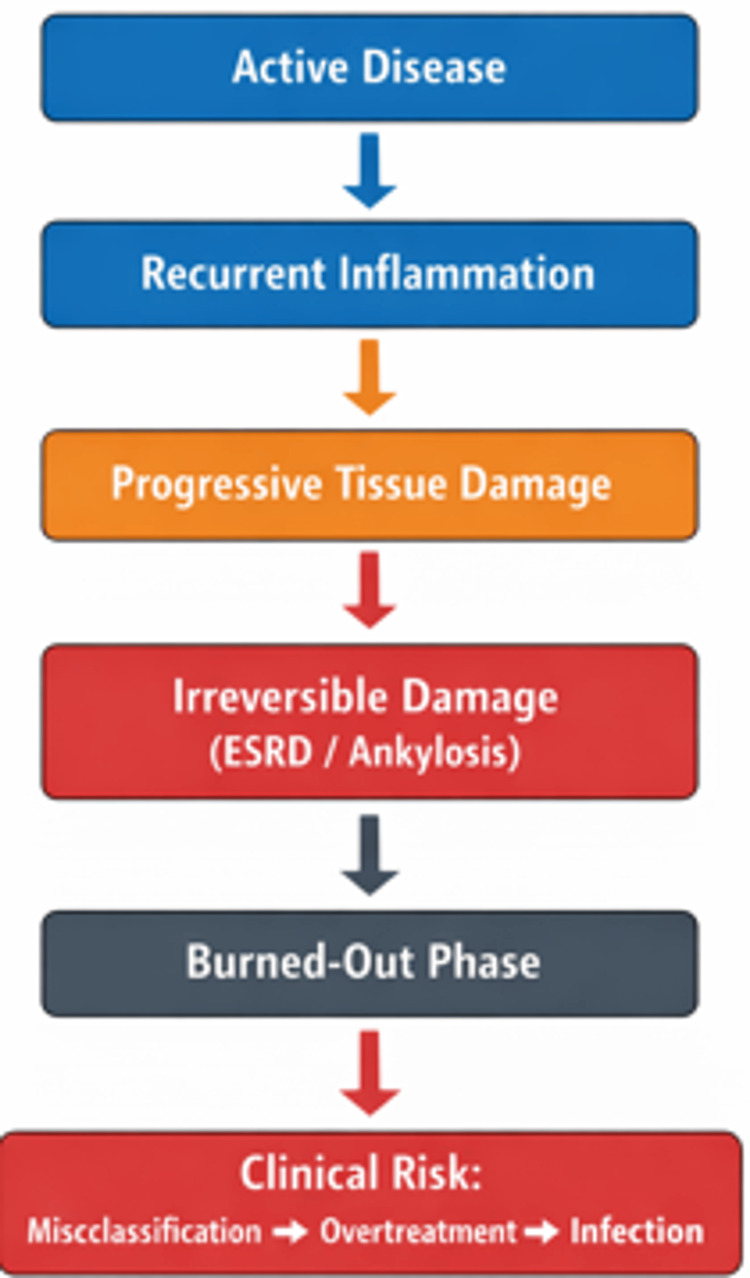
Conceptual model illustrating the transition from active autoimmune disease to a burned-out phase characterized by irreversible structural damage and absence of ongoing inflammation ESRD: end-stage renal disease

**Figure 2:**
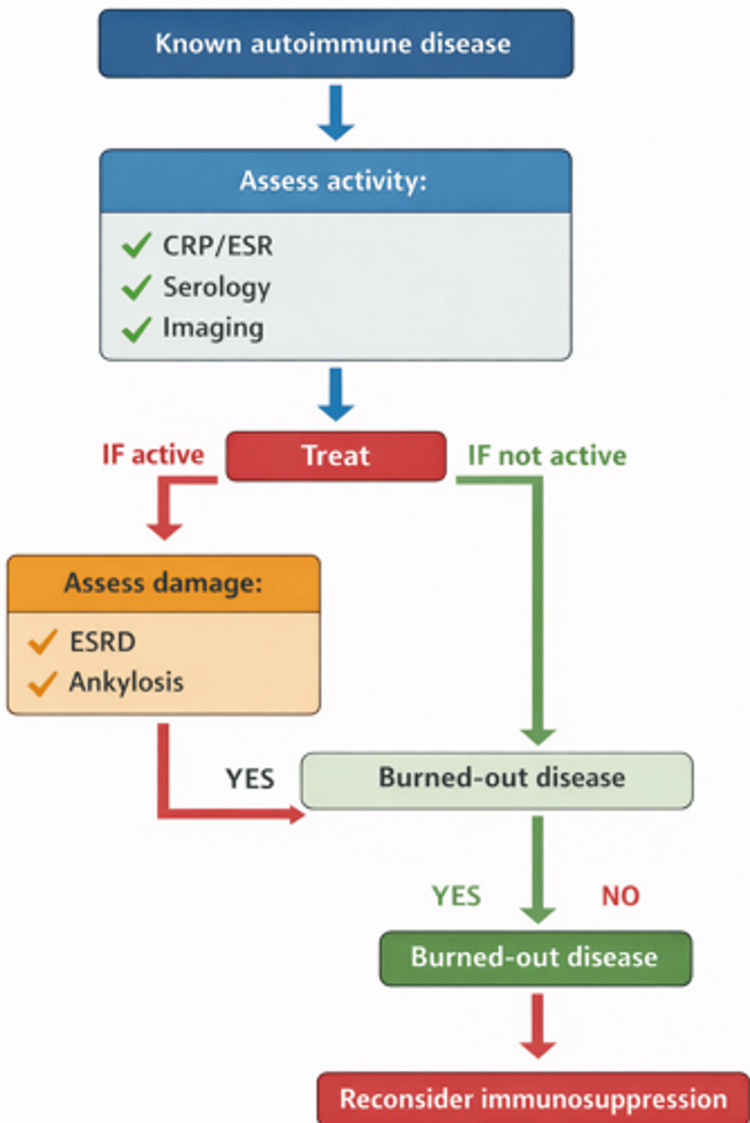
Proposed clinical decision-making algorithm to differentiate active autoimmune disease from burned-out disease and guide treatment decisions CRP: C-reactive protein; ESR: erythrocyte sedimentation rate; ESRD: end-stage renal disease

A conceptual framework describing the proposed burned-out autoimmune disease model and a clinical decision-making algorithm were developed to summarize the study concept and the proposed approach for differentiating active inflammation from irreversible damage (Figures 1-2).

## Results

A total of seven patients with established autoimmune diseases were included in this retrospective multicenter case series. The cohort included patients with ANCA-associated vasculitis, lupus nephritis, medium-vessel vasculitis, and axial spondyloarthritis. All patients demonstrated a prior phase of objectively active inflammatory disease followed by progression to advanced irreversible organ damage, including ESRD, chronic vascular occlusion, or structural ankylosis.

Despite persistent organ dysfunction and chronic symptoms, subsequent follow-up demonstrated sustained clinical and serological quiescence with absent or minimal objective evidence of ongoing inflammatory activity. Disease activity during follow-up was retrospectively evaluated using disease-specific validated measures, including BVAS-based assessment for vasculitis, SLEDAI-based serological assessment for lupus nephritis, and ASDAS/ASAS-based assessment for axial spondyloarthritis, all supporting inactive disease states in the proposed burned-out cases. Continued immunosuppressive therapy in one patient was associated with fatal opportunistic cytomegalovirus infection.

Case 1. ANCA-associated vasculitis (burned-out with fatal infectious outcome)

A 74-year-old female with perinuclear-ANCA (p-ANCA)-associated vasculitis (microscopic polyangiitis) was initially diagnosed at the age of 50 after presenting with diffuse alveolar hemorrhage confirmed by bronchoscopy and rapidly progressive renal failure. Renal biopsy confirmed microscopic polyangiitis. She received induction therapy with rituximab followed by azathioprine maintenance, achieving clinical remission. Over subsequent years, the patient progressed to ESRD requiring long-term hemodialysis. Following the onset of ESRD, there was no clinical, serological, or radiological evidence of active vasculitis, with stable inflammatory markers and absence of pulmonary or systemic relapse. BVAS-based assessment during follow-up was consistent with inactive disease, with no evidence of active pulmonary hemorrhage, constitutional vasculitic manifestations, active urinary sediment, or radiological relapse. 

Despite the absence of objective disease activity, the patient continued on immunosuppressive therapy. Her later clinical course was characterized by frequent hospital admissions (six to seven per year) due to recurrent infections, including pneumonia, urinary tract infections, and gastrointestinal infections. She also developed an ischemic stroke. During her final admission, she presented with bronchopneumonia, cytopenia, and fresh rectal bleeding. Cytomegalovirus (CMV) PCR was markedly elevated (>10,000 IU/mL) in both blood and endotracheal samples, consistent with severe opportunistic CMV infection.

Her condition deteriorated rapidly into septic shock, resulting in death. The clinical course illustrates a transition from active vasculitis to a burned-out state following ESRD, in which ongoing immunosuppression in the absence of active disease may contribute to cumulative immunosuppression burden and severe opportunistic infection.

Case 2. Medium-vessel vasculitis (polyarteritis nodosa-like, burned-out phase)

A 37-year-old Kuwaiti female with poorly controlled type 2 diabetes mellitus initially presented in 2024 with renal impairment. Kidney biopsy demonstrated advanced glomerulosclerosis without evidence of crescentic or active inflammatory lesions, indicating chronic irreversible renal damage. In 2025, she developed a severe systemic illness characterized by constitutional symptoms, including significant weight loss, with markedly elevated inflammatory markers (CRP 100 mg/L, ESR 120 mm/hr). Her presentation included melena, abdominal pain, pleural effusion, metabolic acidosis, and rapidly progressive renal failure culminating in ESRD requiring hemodialysis.

**Figure 3:**
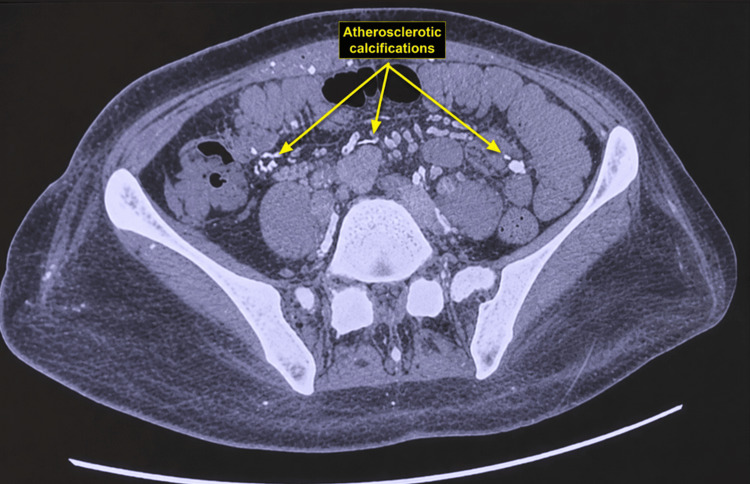
Contrast-enhanced CT abdomen showing chronic atherosclerotic vascular calcifications (arrows) without evidence of active vasculitis CT: computed tomography

CT angiography demonstrated findings consistent with active medium-vessel vasculitis, including celiac and hepatic infarctions, segmental vessel narrowing, and active intra-abdominal bleeding, without evidence of underlying atherosclerosis. She received pulse corticosteroids followed by rituximab (500 mg × two doses, two weeks apart), with subsequent clinical improvement. On follow-up in 2026, the patient reported chronic postprandial abdominal pain. Comprehensive reassessment, including repeat CT angiography, FDG-PET/CT, and arterial Doppler studies, showed no evidence of active vasculitis. Instead, imaging revealed diffuse advanced atherosclerosis involving the abdominal vasculature. Representative follow-up imaging is shown in Figure 3.

Extensive immunological evaluation, including antinuclear antibody (ANA), ANCA, antiphospholipid antibodies, and complement levels, was negative or within normal limits. Repeat imaging and laboratory reassessment supported inactive vasculitic disease without objective evidence of ongoing inflammatory activity.

The clinical course demonstrates an initial phase of active vasculitis followed by transition to a burned-out state characterized by irreversible vascular and renal damage with absence of ongoing inflammatory activity. These findings strongly support the transition from active vasculitis to a damage-dominant state, in which persistent symptoms are driven by irreversible vascular injury rather than ongoing inflammation.

Case 3. Lupus nephritis (severe, juvenile-onset with recurrent activity)

A young male patient was diagnosed with systemic lupus erythematosus (SLE) in 2010 based on arthritis, fever, photosensitivity, and positive antinuclear antibodies. He subsequently developed biopsy-proven class IV lupus nephritis and received induction therapy with cyclophosphamide. In 2014, he experienced a severe disease flare characterized by recurrent class IV lupus nephritis and life-threatening pulmonary hemorrhage requiring intubation and intensive care admission. He was treated with cyclophosphamide and rituximab.

Over the following years, his disease course was marked by recurrent lupus flares with laboratory evidence of activity, including elevated anti-double-stranded DNA levels, hypocomplementemia (low C3 and C4), significant proteinuria exceeding 1.7 g/day, and progressive renal impairment with creatinine rising to 270 µmol/L. These findings were consistent with ongoing active lupus nephritis. Despite maintenance therapy with mycophenolate mofetil, corticosteroids, and hydroxychloroquine, the patient progressed to ESRD in 2023 and was initiated on regular hemodialysis.

Following the onset of dialysis, the patient demonstrated sustained clinical and serological quiescence, with normalization of complement levels, anti-dsDNA titers, and absence of systemic manifestations of lupus activity. Serological and clinical findings were consistent with inactive/low SLEDAI disease activity following dialysis initiation. This case illustrates a transition from highly active, relapsing lupus nephritis to a burned-out disease state following irreversible renal damage.

Case 4. Lupus nephritis (long-standing recurrent disease)

A 48-year-old male with a long-standing history of SLE was diagnosed with biopsy-proven class IV lupus nephritis in 2000 following presentation with rising creatinine and significant proteinuria. His disease course was characterized by recurrent flares of lupus nephritis, supported by laboratory findings including elevated anti-double-stranded DNA levels, low complement levels (C3 and C4), and persistent proteinuria exceeding 2 g/day.

Despite treatment with mycophenolate mofetil and corticosteroids, the patient experienced progressive renal deterioration, ultimately developing ESRD requiring hemodialysis. Within one year of initiating dialysis, the patient exhibited complete clinical and serological inactivity, with normalization of inflammatory markers and complement levels, and absence of active lupus manifestations. He was maintained on hydroxychloroquine alone. Follow-up findings supported sustained inactive lupus disease without evidence of ongoing inflammatory nephritis.

This case further supports the concept of burned-out lupus nephritis in the setting of irreversible renal damage.

Case 5. Lupus nephritis (membranoproliferative pattern)

A female patient with SLE presented with typical clinical and serological features, including photosensitivity, malar rash, positive ANA, elevated anti-double-stranded DNA levels, and hypocomplementemia. She developed significant proteinuria (>2 g/day), and renal biopsy demonstrated a membranoproliferative pattern consistent with lupus nephritis. Despite recurrent disease activity over time, she progressed to ESRD requiring hemodialysis.

Following dialysis initiation, the patient demonstrated sustained clinical and serological remission, with normalization of anti-dsDNA and complement levels and absence of active disease manifestations. Post-dialysis findings were compatible with serologically inactive lupus disease.

This case demonstrates that the burned-out phenomenon may occur across different histopathological subtypes of lupus nephritis.

Case 6. Burned-out ankylosing spondylitis

**Figure 4:**
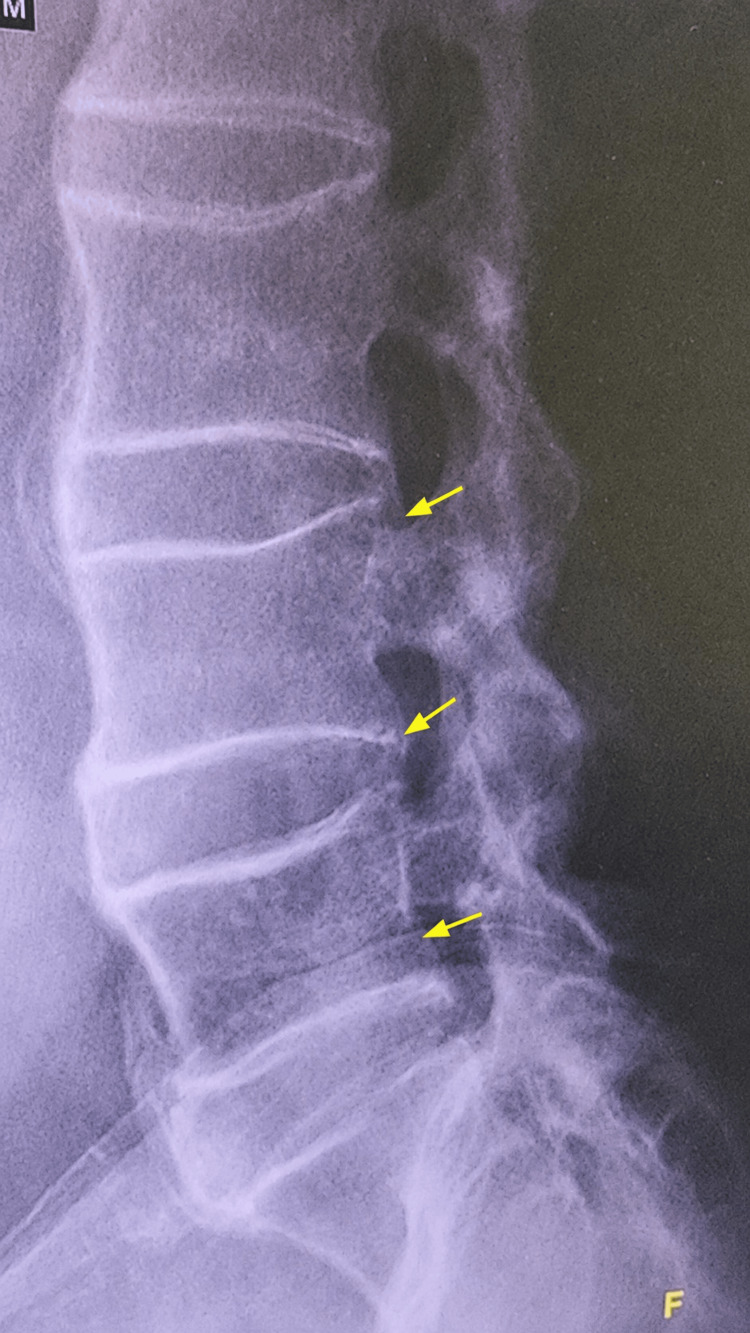
Lateral lumbar spine radiograph demonstrating bridging syndesmophytes and vertebral body squaring (arrows) The findings are consistent with advanced structural damage in axial spondyloarthritis without evidence of active inflammation

**Figure 5:**
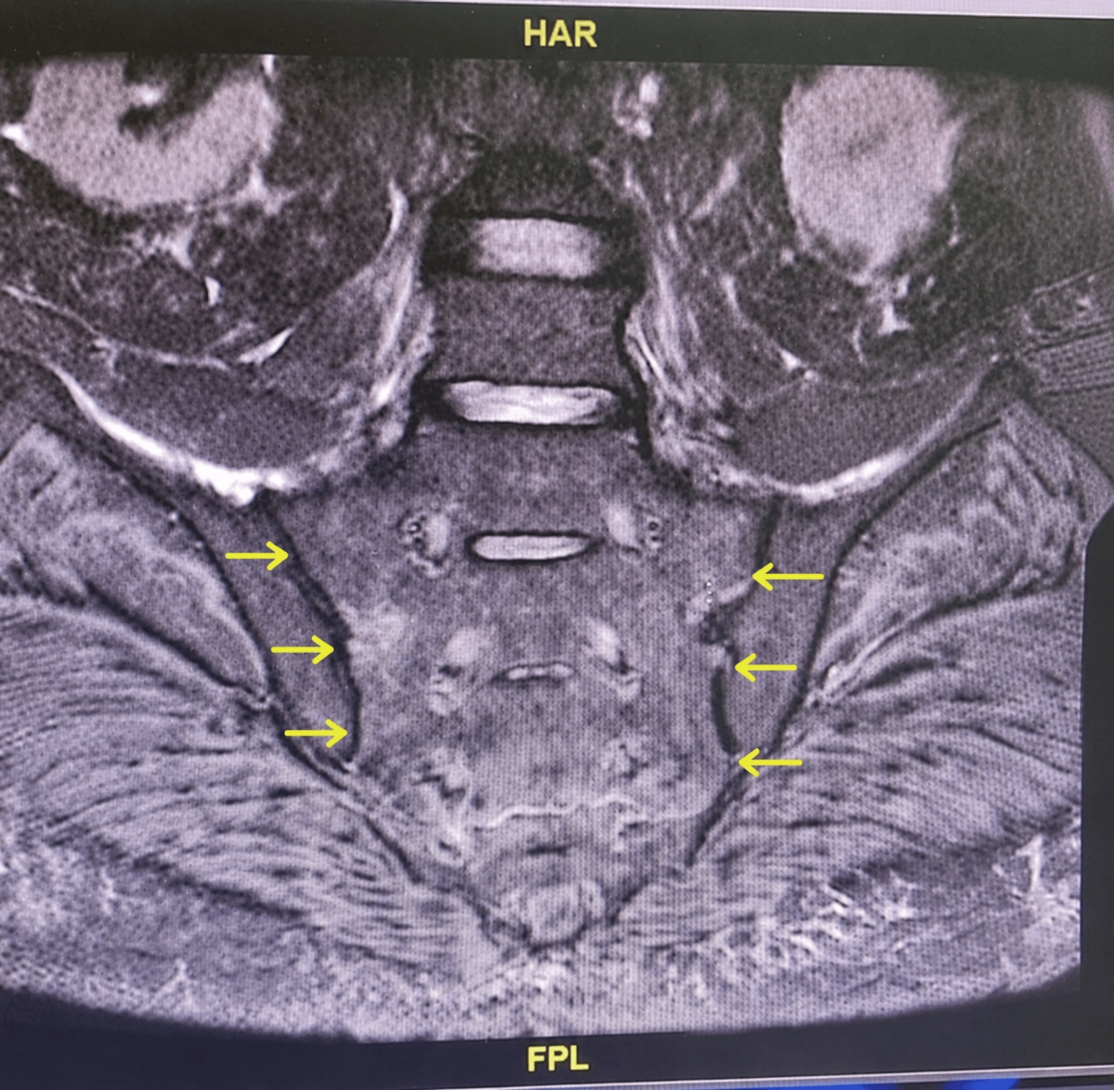
MRI of the sacroiliac joints The image demonstrates chronic structural changes, including erosions and sclerosis at the sacroiliac joints (arrows), without bone marrow edema or active inflammatory features, consistent with inactive (burned-out) axial spondyloarthritis MRI: magnetic resonance imaging

A 64-year-old female with a 20-year history of inflammatory back pain presented with progressive spinal stiffness and functional limitation. She reported no current inflammatory pain. Examination revealed a markedly reduced range of motion of the cervical and lumbar spine without tenderness. Laboratory evaluation showed normal inflammatory markers (CRP 1 mg/L, ESR 11 mm/hr). MRI of the sacroiliac joints demonstrated structural changes, including ankylosis without evidence of bone marrow edema, indicating the absence of active inflammation. ASDAS/ASAS-based assessment supported inactive axial spondyloarthritis with structural damage predominance. Representative radiographic and MRI findings are shown in Figures 4-5.

The findings were consistent with burned-out ankylosing spondylitis, in which structural damage predominates in the absence of ongoing inflammatory activity.

Case 7. Active axial spondyloarthritis (comparison case)

A 35-year-old female presented with a 15-year history of inflammatory back pain associated with worsening symptoms and reduced response to nonsteroidal anti-inflammatory drugs. Clinical examination revealed limited spinal mobility and tenderness over both sacroiliac joints. Laboratory evaluation demonstrated elevated inflammatory markers (CRP 40 mg/L, ESR 50 mm/hr). MRI was not performed due to financial limitations. However, based on the clinical presentation and laboratory findings, she was considered to have active axial spondyloarthritis.

This case serves as a comparator to highlight the distinction between active inflammatory disease and burned-out structural disease.

The clinical characteristics of all included cases are summarized in Table 1.

**Table 1:** Clinical characteristics of included cases AAV: antineutrophil cytoplasmic antibody-associated vasculitis; MPA: microscopic polyangiitis; CMV: cytomegalovirus; PAN: polyarteritis nodosa; CRP: C-reactive protein; ESR: erythrocyte sedimentation rate; CT: CT: computed tomography; ESRD: end-stage renal disease; PET: positron emission tomography; dsDNA: double-stranded DNA; MMF: mycophenolate mofetil; MPGN: membranoproliferative glomerulonephritis; ANA: antinuclear antibody; NSAIDs: nonsteroidal anti-inflammatory drugs; MRI: magnetic resonance imaging

Case	Disease	Age in years/sex	Initial active disease evidence	Treatment during the active phase	Irreversible damage	Evidence of the burned-out state
1	AAV (MPA)	74/F	Diffuse alveolar hemorrhage; biopsy-proven microscopic polyangiitis	Rituximab; azathioprine maintenance	ESRD; long-term hemodialysis	No clinical or serological relapse; recurrent infections; fatal CMV infection
2	PAN-like vasculitis	37/F	Constitutional symptoms; CRP 100 mg/L; ESR 120 mm/hr; CT angiography showing celiac/hepatic infarctions, vessel narrowing, and active bleeding	Pulse corticosteroids; rituximab	ESRD; chronic vascular damage	PET/CT negative; repeat CT angiography negative; serology negative
3	Lupus nephritis (Class IV)	Young/M	Biopsy-proven class IV lupus nephritis; pulmonary hemorrhage; elevated anti-dsDNA; low C3/C4; proteinuria >1.7 g/day	Cyclophosphamide; rituximab; MMF; corticosteroids; hydroxychloroquine	ESRD	Sustained clinical and serological quiescence after dialysis
4	Lupus nephritis (Class IV)	48/M	Recurrent nephritis flares; elevated anti-dsDNA; low C3/C4; proteinuria >2 g/day	MMF; corticosteroids	ESRD	Complete clinical and serological inactivity after dialysis
5	Lupus nephritis (MPGN pattern)	F	Photosensitivity; malar rash; positive ANA; elevated anti-dsDNA; low complement; biopsy showing membranoproliferative pattern	Immunosuppressive therapy	ESRD	Sustained serological remission after dialysis
6	Burned-out axial spondyloarthritis	64/F	Long-standing inflammatory back pain	NSAIDs	Advanced structural spinal damage	MRI without bone marrow edema; normal CRP/ESR
7	Active axial spondyloarthritis	35/F	Inflammatory back pain; sacroiliac tenderness; CRP 40 mg/L; ESR 50 mm/hr	NSAIDs	None	Clinically active disease

Beyond baseline clinical characteristics, differentiation between active inflammatory disease and irreversible damage represented a major challenge across the included cases.

**Table 2:** Distinguishing features between active and burned-out autoimmune disease CRP: C-reactive protein; ESR: erythrocyte sedimentation rate; ANCA: antineutrophil cytoplasmic antibody; dsDNA: double-stranded DNA; BME: bone marrow edema; GN: glomerulonephritis; ESRD: end-stage renal disease

Domain	Active disease	Burned-out disease
Clinical features	Inflammatory symptoms (pain, fever, systemic features)	Mechanical symptoms or stable organ dysfunction
Inflammatory markers	Elevated CRP/ESR	Normal or low
Serology	Active (ANCA, dsDNA, low complement)	Negative or normalized
Imaging	Active inflammation (BME, vasculitis, GN)	Structural damage (ankylosis, fibrosis, infarction)
Organ status	Potentially reversible	Irreversible (ESRD, ankylosis)
Disease course	Relapsing activity	Stable, non-progressive
Response to immunosuppression	Significant improvement	Minimal or no benefit
Risk–benefit of therapy	Benefits outweigh risks	Risks may outweigh benefits

Based on the observed clinical patterns, a practical framework was developed to help identify features supporting a burned-out or damage-dominant autoimmune phenotype. Table 3 was developed by the authors based on observed clinical patterns and a literature review.

**Table 3:** Proposed clinical criteria for burned-out autoimmune disease CRP: C-reactive protein; ESR: erythrocyte sedimentation rate; ESRD: end-stage renal disease

Criterion	Description
Prior confirmed disease	Biopsy, imaging, or serology confirming active autoimmune disease
Absence of objective activity	Normal CRP/ESR, negative serology, no imaging evidence
Irreversible damage	ESRD, ankylosis, fibrosis, infarction
Clinical stability	No relapses over time
Treatment implication	Lack of expected benefit from continued immunosuppression

## Discussion

The present multicenter case series demonstrates a consistent clinical pattern across multiple autoimmune diseases, in which an initial phase of active inflammation transitions into a state characterized by irreversible structural damage and absence of ongoing inflammatory activity. This phenomenon was observed across lupus nephritis, ANCA-associated vasculitis, medium-vessel vasculitis, and axial spondyloarthritis, suggesting a shared cross-disease endpoint despite heterogeneous pathophysiological mechanisms [1-5]. Notably, our fatal case highlights a critical clinical implication of this transition, where continuation of immunosuppressive therapy in the absence of objective disease activity contributed to severe opportunistic infection and mortality [4,6,7].

From a pathophysiological perspective, several mechanisms may explain this transition. First, loss of antigenic target following organ destruction, particularly in ESRD, may reduce immune activation, as the primary site of antigen presentation is no longer functional. Second, prolonged immune activation may lead to immune exhaustion, with diminished effector responses over time. Third, chronic inflammation is progressively replaced by fibrosis and structural remodeling, resulting in persistence of damage without active inflammatory signaling. These mechanisms have been described in vasculitis and chronic glomerular diseases and are biologically plausible across autoimmune conditions [4,7-9].

In ANCA-associated vasculitis, multiple cohort studies have shown that relapse rates significantly decline after progression to ESRD, while infection-related morbidity and mortality increase, reflecting a shift in the risk-benefit balance of immunosuppression [4,7,10,11]. Importantly, ANCA titers alone are not reliable indicators of disease activity, and treatment decisions should not be based solely on serological markers [1,4]. Our fatal case illustrates this clinical dilemma, where the absence of objective disease activity was accompanied by continued immunosuppression, culminating in severe opportunistic infection. This aligns with prior evidence suggesting that infection risk may exceed relapse risk in patients with ESRD [4,7,11].

A similar phenomenon is observed in lupus nephritis. Advanced glomerulosclerosis and chronic kidney damage are associated with reduced immunological activity, particularly following progression to ESRD. Several studies have reported decreased lupus activity after initiation of dialysis, with normalization of complement levels and anti-double-stranded DNA titers in a subset of patients [8,10]. While extra-renal disease may still occur, the overall inflammatory burden is often reduced. Our lupus nephritis cases demonstrate this transition clearly, with repeated flares followed by sustained serological quiescence after ESRD, regardless of initial histological subtype.

In axial spondyloarthritis, the distinction between active inflammation and structural damage is well recognized. Ankylosis and syndesmophyte formation represent the consequence of prior inflammation rather than ongoing disease activity. Current ASAS-the European Alliance of Associations for Rheumatology (EULAR) recommendations emphasize the requirement for objective evidence of inflammation, such as elevated CRP or MRI bone marrow edema, before initiating biologic therapy [11,12]. In the absence of such evidence, symptoms are more likely attributable to mechanical factors or structural damage, and escalation of immunosuppressive therapy may not be beneficial.

The central clinical challenge highlighted by this study is the differentiation between active disease and irreversible damage. Persistent symptoms - such as pain, organ dysfunction, or functional limitation - may be mistakenly interpreted as ongoing inflammation. This misclassification can lead to unnecessary continuation or escalation of immunosuppressive therapy, exposing patients to significant risks without clear benefit [2,3]. Our findings reinforce the need for a structured approach to this distinction, incorporating clinical, laboratory, and imaging data.

Based on the cases presented and existing literature, we propose that burned-out autoimmune disease should be defined by four key elements: (1) documented prior active autoimmune disease, (2) absence of objective inflammatory activity, (3) presence of irreversible organ damage, and (4) clinical stability over time. This framework is consistent with current guideline principles emphasizing objective evidence of inflammation before treatment escalation [1,3,5].

The proposed clinical criteria for burned-out autoimmune disease are presented in Table 3. However, we further extend this concept by explicitly incorporating the role of irreversible damage and its implications for treatment decisions. Importantly, the diagnosis of burned-out disease should only be made after careful exclusion of subclinical or occult activity. This may require appropriate imaging, repeated laboratory assessment, and longitudinal follow-up. In addition, disease-specific factors - such as ANCA subtype in vasculitis or extra-renal manifestations in lupus - should be considered when assessing relapse risk [1,4,6].

This distinction has direct therapeutic implications. Current treatment paradigms in autoimmune diseases are largely driven by the detection of inflammatory activity; however, they provide limited guidance for managing patients with advanced irreversible damage. As a result, clinicians may default to continuation of immunosuppression despite a lack of objective evidence of benefit, highlighting an important gap in current guidelines. The proposed burned-out disease concept should currently be considered a descriptive clinical framework rather than a formally validated disease classification.

This study has several limitations. It is based on a small number of cases and lacks prospective validation. Additionally, the retrospective design may introduce selection bias. However, the consistency of findings across multiple diseases and centers supports the robustness of the observed pattern. Future studies are needed to validate the proposed framework and to develop objective criteria for identifying burned-out disease.

## Conclusions

This study highlights the importance of distinguishing irreversible structural damage from persistent inflammatory activity in advanced autoimmune disease. Across multiple rheumatologic conditions, patients demonstrated sustained clinical quiescence despite severe organ dysfunction and chronic disability resulting from prior inflammatory injury. These observations emphasize that persistent symptoms, abnormal imaging findings, or long-standing organ impairment do not necessarily indicate ongoing active disease. Careful integration of clinical assessment, serological evaluation, imaging, and disease-specific activity measures is essential before escalating immunosuppressive therapy. Recognition of this damage-dominant state may help reduce unnecessary treatment exposure and improve individualized long-term management strategies in complex autoimmune diseases.
